# Preoperative Spinal Sagittal Alignment Affects Improvement of Locomotive Syndrome by Four Years After Total Hip Arthroplasty

**DOI:** 10.7759/cureus.77326

**Published:** 2025-01-12

**Authors:** Naofumi Taniguchi, Tetsuro Ohba, Tetsuya Jinno, Jiro Ichikawa, Satoshi Ochiai, Tetsuo Hagino, Tomoyuki Ashizawa, Shohei Shirakura, Ryousuke Koizumi, Hirotaka Haro

**Affiliations:** 1 Department of Orthopaedic Surgery, University of Yamanashi, Chuo, JPN; 2 Department of Orthopaedic Surgery, University of Yamanashi, Yamanashi, JPN; 3 Department of Orthopaedic Surgery, Dokkyo Medical University Saitama Medical Center, Saitama, JPN; 4 Department of Orthopaedic Surgery, National Hospital Organization (NHO) Kofu National Hospital, Kofu, JPN

**Keywords:** postoperative outcome, preoperative factor, sacral slope, spinal sagittal alignment, the 25-question geriatric locomotive function scale, total hip arthroplasty: tha

## Abstract

Introduction

Most patients with hip osteoarthritis requiring total hip arthroplasty suffer from locomotive syndrome stage 3, which indicates difficulty with mobility and social participation. Although total hip arthroplasty improves their locomotive syndrome stage, some patients remain at locomotive syndrome stage 3 after total hip arthroplasty, despite hip function improvement. Patients with severe hip osteoarthritis may have an abnormal spinal sagittal alignment. This study investigated the influence of preoperative spinopelvic parameters for locomotive syndrome improvement at four years after total hip arthroplasty.

Methods

This retrospective cohort study of a prospectively maintained database included 65 patients who had undergone total hip arthroplasty. Patients were divided into two groups based on whether they showed improvement from Locomotive Syndrome stage 3 at four years postoperatively: improved group (n = 51) and unchanged group (n = 14). Preoperative spinopelvic parameters were compared between the two groups and examined using logistic analysis to determine locomotive syndrome improvement. The cut-off values for preoperative key factors of locomotive syndrome improvement obtained using logistic analysis were determined using receiver operating characteristics analysis.

Results

Preoperative sagittal vertical axis was significantly larger and sacral slope was significantly smaller in the unchanged group than in the improved group. In the logistic regression analysis, preoperative sacral slope and the 25-question Geriatric Locomotive Function Scale (GLFS-25) were identified as factors associated with locomotive syndrome improvement. The receiver operating characteristic analysis showed that the cut-off values of preoperative sacral slope and GLFS-25 for locomotive syndrome improvement were 32.5° and 44.5, respectively.

Conclusions

Among hip osteoarthritis (OA) patients with locomotive syndrome stage 3, those who have small preoperative sacral slope and large preoperative GLFS-25 score may have difficulty improving their postoperative mobility. Therefore, it may be useful to suggest preoperatively that such patients should be prepared to use social services and other services after surgery to support their postoperative mobility.

## Introduction

The Japanese Orthopedic Association (JOA) has proposed a definition for a condition termed locomotive syndrome (LS), considering Japan’s super-aged society. LS is a condition of decreased mobility, such as difficulty in walking or climbing stairs. LS, which is defined by the LS risk test, consists of a performance test (the stand-up and two-step tests) and patient-reported outcome measures (the 25-question Geriatric Locomotive Function Scale (GLFS-25)), is categorized into stages 1, 2, or 3. LS stage 1 represents an initial decline in mobility, while LS stage 2 represents a progressive decline in mobility. LS stage 3 is a state of progressive decline in mobility and social participation, which increases the risk of patients’ inability to live independently and the requirement of nursing care. LS occurs because of aging and various musculoskeletal disorders [1-4]. Previous studies have reported that all patients with hip osteoarthritis (OA) requiring total hip arthroplasty (THA) had LS, and many of them were categorized as stage 3 [4, 5].

Impaired mental health status is frequently observed in patients who suffer from pain and functional disorders [6]. It is reported that one-third of hip OA patients showed depression symptoms preoperatively [7]. THA has been successfully used for hip joint reconstruction in patients with hip OA [8]. A strong relationship was observed between mental health recovery and improvements in both pain and functional ability after THA [9]. Several studies have reported that patients with hip OA who undergo THA recover their physical and mental functions after surgery [2, 8, 9]. However, some patients do not regain sufficient physical and mental function, even if their hip pain improves [10-12], and almost half of patients still have LS after THA [1, 2, 5].

In Japan, developmental hip dysplasia is the major cause of severe hip OA requiring THA [13]. Patients with severe hip OA may have an abnormal spinal sagittal alignment [14, 15], which adversely affects gait [16]. Further, THA has been reported to improve spinal sagittal balance by reducing the sagittal vertical axis (SVA) within one year after THA [17-19].

It is imperative for patients with LS stage 3 due to hip OA who have difficulty with mobility and social participation to improve their LS stage after THA. We, therefore, investigated whether preoperative spinopelvic parameters were associated with preoperative and four-year postoperative LS risk test scores in patients with preoperative LS stage 3. Next, we clarified the preoperative spinopelvic parameters that predicted the improvement of LS stage 3 by four years after THA.

## Materials and methods

This study has been approved by the authors’ affiliated institutions, and all participants provided written informed consent.

Patients

A total of 134 patients who underwent primary THA for hip OA between 2016 and 2019 underwent the LS risk test preoperatively in our hospital. We excluded 16 patients with preoperative LS stage 1 or 2. Furthermore, 45 patients had no LS risk test at four years postoperatively and eight patients who underwent another surgery for spinal or lower extremity disorders during the study period were excluded. Ultimately, 65 patients were included in this study.

**Figure 1:**
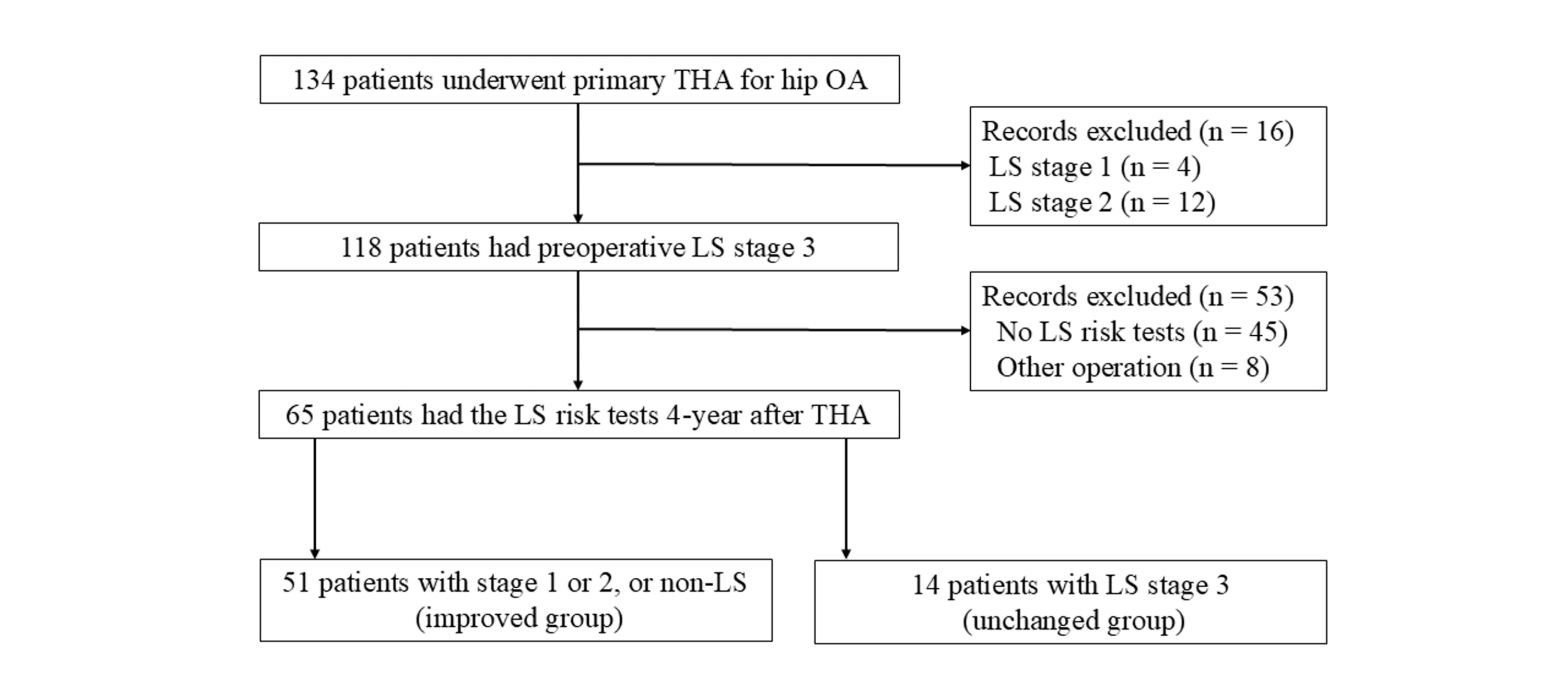
Flowchart of the selection process of participants and their group assignment THA, total hip arthroplasty; OA, osteoarthritis; LS, locomotive syndrome

Patients with preoperative LS stage 3 were divided into two groups based on whether their LS improved at four years postoperatively as those with a change in their LS stage (from stage 3 to stage 1 or 2, or non-LS; improved group) or those with no change in LS stage 3 (unchanged group) (Figure 1).

Surgery and postoperative protocol

All patients underwent THA via an anterolateral approach, performed by an experienced orthopedic surgeon. We performed cementless THA using a 32-mm-diameter ceramic femoral head. All patients got out of bed and started walking exercises with full weight-bearing, as tolerated, the day after surgery. No cases of THA dislocation occurred during the study period.

Outcome measures

Clinical data, including patient characteristics (age, sex, and body mass index [BMI]), disease, JOA hip score, each LS risk test score, and radiographic spinopelvic parameters, were collected through a retrospective review of a prospectively maintained database. The maximum JOA hip score is 100 points and consists of four parameters: pain, range-of-motion (ROM), gait ability, and activities of daily life (ADL).

LS stage was evaluated using the LS risk test, which consisted of a stand-up test, two-step test, and the GLFS-25. The stand-up test measures the strength of lower limb by testing a patient’s ability to rise up from various heights (ranging from 40 to 10 cm in 10 cm decrements). This test is performed first using both legs standing (BLS) and then with a single leg standing (SLS) [20]. The assessment protocol progresses systematically through eight levels of increasing challenge: BLS from 40 cm (minimum difficulty), BLS from 30 cm, BLS from 20 cm, BLS from 10 mc, SLS from 40 cm, SLS from 30 cm, SLS from 20 cm, SLS from 10 cm (maximum difficulty). The stand-up test utilizes a 9-level scoring system ranging from 0 to 8. Participants who cannot stand up with BLS from 40 cm receive a score of 0, while those who successfully stand up with SLS from 10 cm earn a score of 8. The participant's final score corresponded to the most challenging level they could successfully complete.

The two-step test serves as a comprehensive measure of gait functionality, incorporating elements of postural control, lower extremity strength, and range of motion. The protocol requires participants to execute two consecutive steps at their maximum achievable length. The resultant score is calculated as a ratio: the combined length of two maximal strides (measured in centimeters) normalized to the participant's height (also in centimeters) [20].

The GLFS-25 consists of 25 items, including four questions (Q1-4) about body pain in the past month and 21 questions (Q5-25) about usual daily activities including social activities and mental health in the past month. Each item is evaluated using a graduated scale from 0 to 4, where 0 represents a complete absence of functional limitation and 4 indicates profound impairment. The total score ranged from 0, indicating optimal function without syndromes, to 100, representing maximum symptom severity and disability.

The JOA has defined a three-tiered classification system for LS severity based on the LS risk test score.

Classification criteria for LS stage 1 include any of the following conditions:

Stand-up test performance below 5 points (unable to rise from a 40-centimeter platform using one leg)

Two-step test ratio falling between 1.1 and 1.3

GLFS-25 questionnaire score reaching or exceeding 7 points

Classification criteria for LS stage 2 include any of the following conditions:

Stand-up test score below 3 points (inability to rise from a 20-centimeter platform using both legs)

Two-step test ratio ranging from 0.9 to 1.1

GLFS-25 assessment score at or above 16 points

The most severe classification, Stage 3, is determined by any of the following criteria:

Stand-up test performance below 2 points (cannot rise from a 30-centimeter platform using both legs)

Two-step test ratio falling below 0.9

GLFS-25 evaluation score equaling or surpassing 24 points

All participants in this study underwent whole-spinal lateral radiography in the standing position during preoperative and the four years postoperative visits. The spinopelvic parameters measured were thoracic kyphosis, lumbar lordosis (LL), global tilt (GT), SVA, pelvic tilt (PT), sacral slope (SS), pelvic incidence (PI), and pelvic incidence minus lumbar lordosis (PI - LL).

Statistical analysis

All data are expressed as means (standard deviations). Differences in age, BMI, JOA hip score and its parameters, the LS risk test scores, LS stage, and spinopelvic parameters between the unchanged and improved groups were assessed using Welch’s t-test. Sex differences between the groups were assessed using Pearson’s chi-square test. JOA hip score, each parameter of JOA hip score, each of the LS risk test scores and LS stage were compared preoperatively and at four years postoperatively using the Wilcoxon signed-rank test. The relationships between the preoperative spinopelvic parameter values and each four-year postoperative LS risk test score were analyzed using Pearson’s correlation analysis. Preoperative factors affecting LS improvement were identified in different preoperative items between the unchanged and improved groups using logistic regression analysis, and their cut-off values were identified as the maximum point of the Youden index using receiver operating characteristic curves. Statistical analyses were performed using the IBM SPSS Statistics version 27 (IBM Corp., Armonk, NY, USA). Statistical significance was set at p < 0.05.

## Results

The preoperative age, age range, sex, and BMI of patients in the unchanged (n = 14) and improved (n = 51) groups are shown in Table 1. The unchanged group tended to be older than the improved group (p = 0.053). The sex ratio and BMI were not significantly different between the two groups.

**Table 1:** Preoperative patient characteristics Values represent the mean (standard deviation) Differences in age and body mass index between both groups were assessed using Welch’s t-test. Sex differences between the groups were assessed using Pearson’s chi-square test. *chi-square value Statistical significance was set at p < 0.05 BMI, body mass index; OA, osteoarthritis.

	All	Unchanged group	Improved group	t value	p value
Number	65	14	51		
Age (years)	67.3 (11.2)	73.0 (11.8)	65.8 (10.6)	2.065	0.053
Age range (years)	38 - 84	46 - 84	38 - 83		
Sex (female/ male)	58/ 7	12/ 2	46/ 5	0.230*	0.632
BMI (kg/m^2^)	23.8 (4.1)	25.4 (5.2)	23.3 (3.7)	1.389	0.183
Disease	Dysplastic hip OA				

Table 2 shows the preoperative and four years postoperative JOA hip score, its parameters, each score of the LS risk test and LS stage. The JOA hip score, gait ability, and each of the LS risk test scores of the improved group were significantly better than those of the unchanged group preoperatively. Four years postoperatively, the improved group was significantly better than the unchanged group on all items except pain and ROM. Comparing preoperative and four years postoperative results, the improved group showed improvement in all items and the unchanged group did not improve in ADL and each of the LS risk test.

**Table 2:** The JOA hip score, its parameters, and each of the locomotive syndrome risk test score preoperatively and four years postoperatively Values represent the mean (standard deviation) Differences in JOA hip score, each parameter of JOA hip score, each of the LS risk test scores, and LS stage between both groups were assessed using Welch’s t-test. JOA hip score, each parameter of JOA hip score, each of the LS risk test scores, and LS stage were compared preoperatively and at four years postoperatively using the Wilcoxon signed-rank test. Statistical significance was set at p < 0.05. * Significant difference compared to corresponding items preoperatively and four years postoperatively. JOA, Japanese Orthopaedic Association; ROM, range of motion; ADL, activities of daily living; GLFS-25, 25-question Geriatric Locomotive Function Scale; LS, locomotive syndrome

	All	Unchanged group	Improved group	t value	p value
Preoperative					
JOA hip score	55.4 (10.2)	49.6 (9.2)	56.1 (9.3)	-2.325	0.030
Pain	18.0 (5.4)	17.1 (4.7)	18.2 (5.2)	-0.755	0.458
ROM	12.0 (6.7)	12.1 (2.5)	12.0 (3.0)	0.205	0.839
Gait ability	11.4 (4.4)	8.2 (4.2)	12.3 (4.0)	-3.273	0.004
ADL	13.1 (4.4)	12.1 (1.9)	13.4 (2.2)	-1.987	0.059
Stand-up test	2.2 (1.4)	1.4 (1.1)	2.3 (1.4)	-2.634	0.014
Two-step test	0.9 (0.3)	0.7 (0.4)	1.0 (0.2)	-2.288	0.036
GLFS-25	46.4 (17.1)	54.4 (16.7)	42.9 (15.1)	2.396	0.031
LS stage	3 (0)	3 (0)	3 (0)	6554	1.000
4 years postoperative					
JOA hip score	92.4 (7.2)*	85.2 (11.6)*	94.3 (4.4)*	-3.635	0.003
Pain	39.8 (1.1)*	40.0 (0)*	39.6 (1.3)*	1.768	0.083
ROM	17.2 (2.2)*	16.7 (2.3)*	17.1 (2.0)*	-1.106	0.283
Gait ability	18.0 (3.8)*	14.1 (6.1)*	19.3 (1.6)*	-3.871	0.002
ADL	17.4 (2.8)*	14.4 (2.3)	18.4 (1.9)*	-3.528	0.003
Stand-up test	3.2 (1.4)*	1.8 (1.1)	3.6 (1.2)*	-5.283	< 0.001
Two-step test	1.2 (0.3)*	0.8 (0.4)	1.3 (0.2)*	-4.777	< 0.001
GLFS-25	13.9 (19.1)*	42.4 (24.4)	6.1 (4.8)*	5.522	< 0.001
LS stage	1.5 (1.1)	3 (0)	1.1 (0.8)*	16.845	< 0.001

A comparison of the preoperative spinopelvic parameters between the unchanged and improved groups is shown in Table 3. Preoperative SVA was significantly larger and preoperative SS was significantly smaller in the unchanged group than in the improved group.

**Table 3:** Comparison of spinopelvic parameters between the unchanged and improved groups preoperatively Values represent the mean (standard deviation) Differences in spinopelvic parameters between both groups were assessed using Welch’s t-test. Statistical significance was set at p < 0.05 TK, thoracic kyphosis; LL, lumbar lordosis; GT, global tilt; SVA, sagittal vertical axis; PT, pelvic tilt; SS, sacral slope; PI, pelvic incidence.

	Unchanged group	Improved group	t value	p value
TK	26.6 (17.5)	28.1 (8.6)	-0.318	0.755
LL	26.0 (21.0)	37.1 (14.5)	-1.857	0.081
GT	31.3 (23.4)	21.1 (15.0)	1.562	0.138
SVA	62.8 (53.6)	28.6 (38.0)	2.120	0.049
PT	24.7 (16.9)	18.0 (12.0)	1.389	0.183
SS	25.7 (12.9)	34.8 (9.1)	-2.468	0.025
PI	50.4 (12.5)	52.8 (11.5)	-0.639	0.530
PI -LL	24.4 (26.7)	15.7 (16.4)	1.161	0.263

Some preoperative spinopelvic parameters were significantly correlated with the four-year postoperative performance tests (stand-up and two-step tests); however, no significant correlation was found between the preoperative spinopelvic parameters and preoperative GLFS-25 scores (Table 4). Preoperative GT, SVA, and PT were significantly correlated with the four-year postoperative stand-up test results. In addition to the preoperative spinopelvic parameters, which were significantly correlated with the preoperative two-step test results, the preoperative SVA and SS were also significantly correlated with the four-year postoperative two-step test results. The preoperative LL, GT, SVA, PT, SS, and PI-LL showed significant correlations with the four-year postoperative GLFS-25 results (Table 4).

**Table 4:** Correlation between preoperative spinopelvic parameters and locomotive syndrome risk test scores in all patients The relationships between the preoperative spinopelvic parameter values and each four-year postoperative LS risk test score were analyzed using Pearson’s correlation analysis. Statistical significance was set at p < 0.05. * Significant difference as compared to the respective preoperative spinal parameters in both groups (p < 0.05) GLFS-25, 25-question Geriatric Locomotive Function Scale; TK, thoracic kyphosis; LL, lumbar lordosis; GT, global tilt; SVA, sagittal vertical axis; PT, pelvic tilt; SS, sacral slope; PI, pelvic incidence.

	Preoperative				4 Years Postoperative		
	Stand-up test	Two-step test		GLFS-25	Stand-up test	Two-step test	GLFS-25
TK	–0.119	0.072	–0.074		–0.244	–0.079	–0.054
LL	–0.077	0.063	–0.076		0.158	0.299*	–0.332*
GT	–0.305*	–0.273*	0.043		–0.321*	–0.360*	0.343*
SVA	–0.285*	–0.240	0.134		–0.323*	–0.345*	0.376*
PT	–0.282*	–0.263*	–0.038		–0.255*	–0.319*	0.297*
SS	–0.043	0.112	0.079		0.240	0.370*	–0.282*
PI	–0.362*	–0.200	0.028		–0.072	–0.028	0.083
PI - LL	–0.154	–0.176	0.083		–0.181	–0.275*	0.337*

Based on the results of Tables 1-3, logistic regression analysis was performed using preoperative factors such as age, JOA hip score, Stand-up test, Two-step test, GLFS-25, SVA and SS as explanatory variables for LS improvement (Table 5).

**Table 5:** Logistic regression analysis results of spinopelvic parameters associated with locomotive syndrome improvement Preoperative factors affecting LS improvement were identified using logistic regression analysis. Statistical significance was set at p < 0.05. SE, standard error; OR, odds ratio; CI, confidence interval; SS, sacral slope; GLFS-25, 25-question Geriatric Locomotive Function Scale

	β	SE	OR (95% CI)	p
SS	0.108	0.038	1.114 (1.034, 1.201)	0.005
GLFS-25	-0.063	0.023	0.939 (0.897, 0.983)	0.007

**Figure 2:**
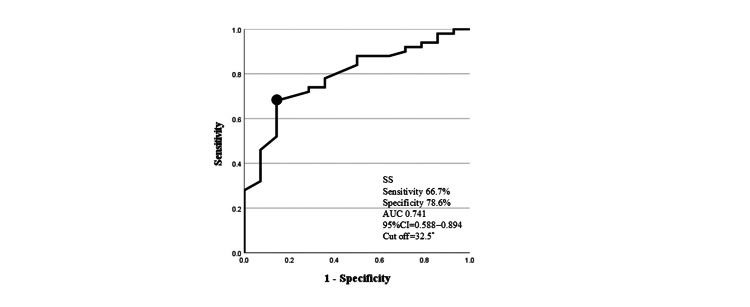
ROC curves for prediction of improvement in locomotive syndrome by SS. Area under the ROC curve, 0.741 (95% CI: 0.588–0.894); sensitivity, 66.7%; and specificity, 78.6% at a cut-off of 32.5°. SS, sacral slope; ROC, receiver operating characteristic; CI, confidence interval

**Figure 3:**
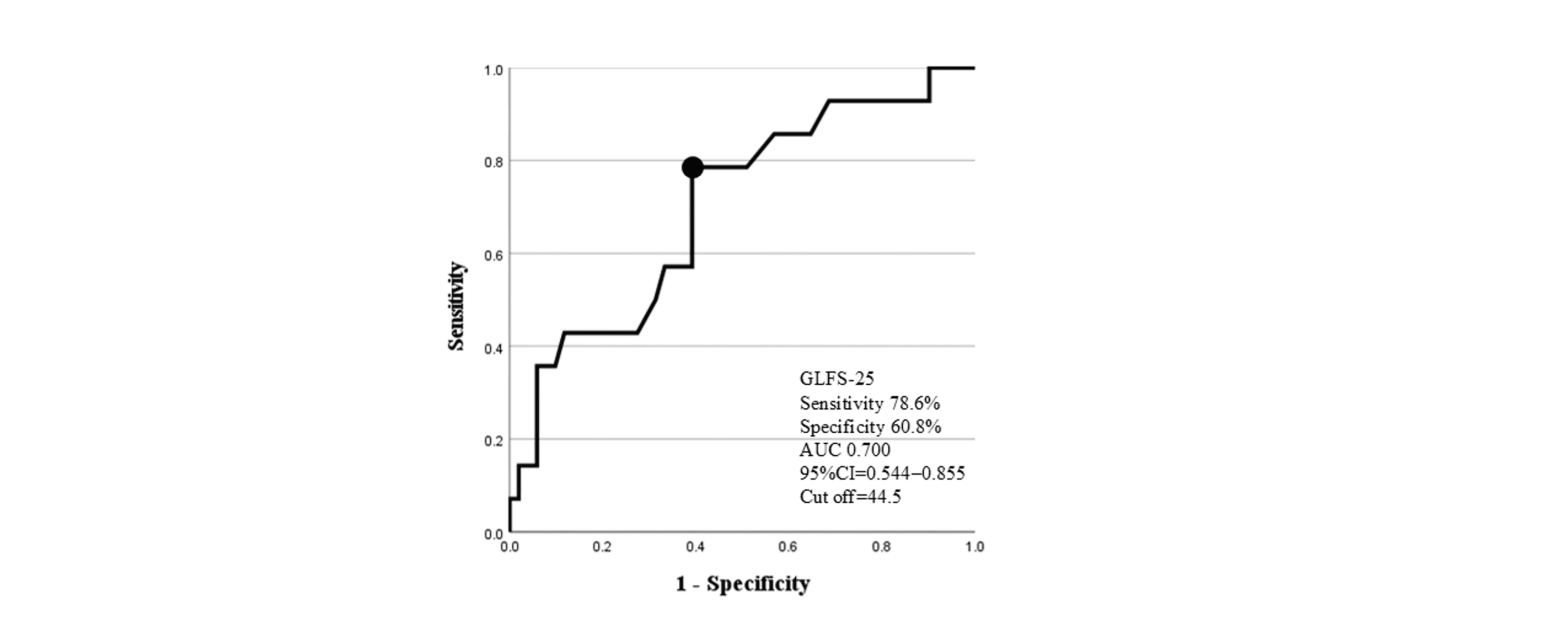
ROC curves for prediction of improvement in locomotive syndrome by GLFS-25. Area under the ROC curve, 0.700 (95% CI: 0.544–0.855); sensitivity, 78.6%; and specificity, 60.8% at a cut-off of 44.5. GLFS-25, the 25-question Geriatric Locomotive Function Scale; ROC, receiver operating characteristic; CI, confidence interval.

Preoperative SS and GLFS-25 were detected as factors related to improvement from LS stage 3 by four years after THA. The cut-off values of preoperative SS and GLFS-25 for improvement of LS were 32.5° and 44.5, respectively (Figures 2, 3).

## Discussion

Several studies have reported that treatments for various diseases improve LS stage, which reflects mobility [1-3, 21]. Recently, some studies have reported postoperative progress in LS stage in patients who have undergone THA [1, 4, 22]. Of patients with hip OA with preoperative LS stage 3, 61.5% (67/109) - 75.6% (59/78) had improved LS after THA [4, 22]. Higher age, higher BMI, and lower functional reach test scores have been reported as preoperative factors predicting LS improvement after surgery [1, 4, 22]. However, one-third to one-fourth of patients do not show improved LS after THA [4, 22]. In the current study, the LS stage of 21.5% (14/65) of patients had not improved four years after THA. We considered it important to investigate preoperative factors other than those reported previously. To our knowledge, no studies have evaluated whether preoperative spinopelvic parameters influence the improvement of LS four years after THA. Evaluating improvement in patients with LS stage 3 following THA is a significant research topic with substantial implications for sociomedical economics. Then, we investigated whether preoperative spinopelvic parameters were related to improvement from LS stage 3 by four years after THA. This study revealed that improvement in LS stage 3 could be predicted based on preoperative spinal parameters, which is clinically important.

Previous studies have reported that preoperative spinopelvic parameters affect the outcomes after THA. Ochi et al. reported that patients with imbalanced preoperative sagittal alignment, such as a larger SVA and larger PI-LL, had worse clinical outcomes than those who underwent THA [23]. Okamoto et al. reported that the preoperative T1 pelvic angle divided by the PI was associated with greater disability (hip disability and OA outcome core joint replacement < 70/100) by two years after THA [24]. Okuzu et al. reported that the LL, SVA, and PI-LL were key factors influencing low back pain by one year after THA [25]. Nagatani et al. reported SS as a risk factor for progressive spinal sagittal imbalance by three years after THA [26]. The current study showed that preoperative SS and GLFS-25 affected improvement from LS stage 3 by four years after THA. Generally, LL and SS become smaller, and SVA becomes larger, leading to a progressive spinal sagittal imbalance, which adversely affects gait. The results of this study suggested that smaller preoperative SS adversely affects the improvement of LS after THA.

Sagittal spinopelvic parameters affect stand-up and two-step test scores [27, 28]. Although preoperative SVA and SS were not correlated with the preoperative two-step test in the present study, these preoperative spinopelvic parameters correlated significantly with the results of the four-year postoperative two-step test, which evaluates walking ability (including balance, leg muscle strength, and flexibility of the lower extremities) (Table 4). Although preoperative hip pain and restricted ROM might have masked the impact of spinal alignment on the preoperative two-step test, the effect of preoperative sagittal spinopelvic alignment on the two-step test was more apparent after THA. The results of the current study suggested that a high GLFS-25 score is an inhibiting factor for LS improvement after THA. While no correlation was found between spinopelvic parameters and GLFS-25 preoperatively, preoperative LL, GT, SVA, PT, SS, and PI-LL were associated with GLFS-25 four years after THA in this study. Associations between several spinopelvic parameters, including SVA, PT, SS, PI-LL, and GLFS-25, have been reported in adult patients with spinal deformities [29]. The association between preoperative spinopelvic parameters and the four-year postoperative GLFS-25 score in this study reflects the effect of spinal sagittal alignment on LS in patients who have undergone THA.

The current study had some limitations that should be acknowledged. First, the sample size was small. Data were collected prospectively; therefore, participants who did not undergo the LS risk test were excluded. Further studies with larger sample sizes are needed to ensure that the LS risk test data are not missing for participants. Second, the current study did not properly match participants between the groups. Spinopelvic parameters and LS are affected by age, and the preoperative LS risk test differed significantly between the two groups. We believe that comparisons should rather be done after age-matching of the groups. However, the population in the unchanged group was smaller than that in the improved group. Therefore, further studies with larger sample sizes are warranted. Third, although we performed multivariate analyses incorporating age, JOA hip score, and LS risk test results, we were unable to include all potential confounding factors in our analyses. Finally, we did not assess joint diseases other than hip joint diseases, as the data in this study population specifically excluded individuals with severe joint diseases necessitating surgical treatment.

## Conclusions

Nearly 80% (51/65) of patients with LS stage 3 who underwent THA due to hip OA showed improved LS stage by four years after THA. Preoperative SS and GLFS-25 were identified as factors associated with LS improvement. Preoperative assessment of spinopelvic alignment is thus useful for predicting the postoperative course of LS. Patients with small preoperative SS and large GLFS-25 score adversely affected the improvement in LS and may have difficulty improving their postoperative mobility after THA. Therefore, it may be useful to suggest preoperatively that such patients should be prepared to use social services and other services after surgery to support their postoperative mobility.
